# 

**Published:** 1944

**Authors:** 


### CONTENTS.

| | Page |
|---|---|
| **The Thirty-Second Long Fox Memorial Lecture: The Social Aspects of Acute Rheumatism.** By Professor C. Bruce Perry, M.D., F.R.C.P. | 1 |
| **"Look and See," Foreign Bodies in Air and Food Passages.** By E. Watson-Williams, M.C., Ch.M., F.R.C.S. | 11 |
| **Edward Tyson, M.D., F.R.S., 1650–1708** | 15 |
| **The Goodenough Report on Medical Education** | 17 |
| **Reviews of Books** | 21 |
| **Editorial Notes** | 26 |
| **Obituaries:** Captain Edward Mills Grace, M.B., Ch.B., R.A.M.C. | 27 |
| Major Leslie R. Jordan, M.B., Ch.B., R.A.M.C. | 27 |
| Frederic Percival Mackie, C.S.I., O.B.E., M.D., M.Sc., F.R.C.S. | 28 |
| Frederick John Poynton, M.D., F.R.C.P. | 29 |
| Francis Wilfred Willway, M.D., M.S., F.R.C.S. | 30 |
| **The Medical Library of the University of Bristol** | 33 |
| **Publications Received** | 35 |
| **Local Medical Notes** | 36 |

